# Research progress on the antitumor immune regulation mechanisms and combined treatment strategies of histotripsy: A review

**DOI:** 10.1097/MD.0000000000049082

**Published:** 2026-06-05

**Authors:** Xv Sun, Fanbo Wang

**Affiliations:** a Clinical Medical College of Jiamusi University, Jiamusi, Heilongjiang, China; b Department of Physical Diagnosis, The First Affiliated Hospital of Jiamusi University, Jiamusi, Heilongjiang, China.

**Keywords:** abscopal effect, antitumor immunity, combined therapy, hepatocellular carcinoma, histotripsy, immunomodulatory mechanism

## Abstract

Histotripsy is a noninvasive, nonionizing, nonthermal ablation technology approved by the US FDA for liver cancer treatment in 2023. It exerts antitumor effects via focused ultrasound cavitation, with unique immunomodulatory advantages. Core mechanisms include inducing immunogenic cell death to form an “in situ vaccine,” reshaping tumor microenvironment immune cell infiltration, regulating cytokine networks, and triggering a CD8 + T cell-mediated ferroptosis-related “abscopal effect.” Combined with immune checkpoint inhibitors, antiangiogenic drugs, or chemotherapy, it shows significant synergistic efficacy. Clinical trials such as #HOPE4LIVER confirm its 95% technical success rate in hepatocellular carcinoma. With further optimization, Histotripsy is expected to benefit more solid tumor patients.

## 1. Introduction

### 1.1. Overview of histotripsy technology

As a revolutionary noninvasive technology in the field of local tumor therapy, Histotripsy achieves mechanical tissue destruction through focused ultrasound cavitation, boasting unique advantages of noninvasiveness, nonionization, and nonthermal ablation.^[1]^ Unlike traditional thermal ablation techniques, it liquefies target tissues into acellular homogeneous debris via precisely controlled acoustic cavitation, avoiding thermal damage to surrounding normal tissues throughout the process.^[2]^

The core principle of this technology lies in the cavitation effect induced by high-intensity focused ultrasound: ultrasonic energy generates microbubbles at the focal point, and the mechanical stress produced by the rapid expansion and contraction of these bubbles damages cell membranes and intracellular structures, ultimately achieving precise liquefaction of target tissues.^[3]^ In October 2023, the US FDA approved Histotripsy for the treatment of liver tumors, marking the successful transformation of this technology from laboratory research to clinical application and providing a novel noninvasive option for liver cancer treatment.^[4]^

### 1.2. Interaction between local tumor therapy and immune regulation

Advances in tumor immunology research have promoted a shift in therapeutic concepts from “simple tumor killing” to “equal emphasis on killing and immune activation.” Local therapy destroys tumor tissues to release tumor-associated antigens and damage-associated molecular patterns (DAMPs), which activate antigen-presenting cells and further induce specific antitumor immune responses.^[5]^

Traditional thermal ablation techniques tend to cause antigen denaturation, while nonthermal ablation technologies such as Histotripsy can better preserve the natural conformation of antigens, producing a stronger immune activation effect.^[6]^ The immunosuppressive microenvironment commonly existing in solid tumors (e.g., regulatory T cell infiltration, overexpression of immune checkpoint molecules, hypoxia, etc) limits therapeutic efficacy. However, local therapy can reshape the tumor microenvironment, potentially converting “cold tumors” into “hot tumors” and improving the sensitivity to subsequent immunotherapy.^[7]^

### 1.3. Immune activation advantages of histotripsy compared to traditional ablation

The immune activation advantages of Histotripsy stem from its nonthermal mechanical destruction mechanism: first, it can preserve the immunogenicity of tumor antigens, and the tumor homogenate it produces contains peptide antigens with immune activity, which is not observed in cryo-thermal liquefaction products^[2]^; second, it can induce typicalimmunogenic cell death (ICD), releasing DAMPs such as calreticulin (CRT) and high mobility group box 1 (HMGB1) to activate dendritic cells (DCs) and T cells^[1]^; third, it has a stronger ability to recruit immune cells – after treatment, the number of innate and adaptive immune cells such as CD8 + T cells, NK cells, and DCs in tumors increases significantly, and it can induce the production of functional tumor-specific CD8 + T cells^[1]^; fourth, it can produce a significant “abscopal effect” – unilateral treatment can inhibit the growth of untreated contralateral tumors and prolong survival, which is closely related to systemic antitumor immune responses.^[1,8]^

## 2. Methods

### 2.1. Study design

This study is a narrative review focusing on the antitumor immune regulation mechanisms and combined treatment strategies of histotripsy. We followed the PRISMA guidelines to standardize literature search, study selection, and data extraction for transparency.

### 2.2. Literature search and study selection

A systematic literature search was conducted in multiple databases (PubMed, Web of Science, Embase, Cochrane Library) with keywords: histotripsy, antitumor immunity, immune regulation, combined therapy, hepatocellular carcinoma, abscopal effect. Two reviewers independently screened literature, extracted data, and resolved discrepancies via consultation.

## 3. Results

This narrative review synthesized the key preclinical and clinical findings regarding histotripsy as follows. Histotripsy exerts multi-level antitumor immunomodulatory effects and exhibits favorable clinical efficacy in liver tumors, with promising synergistic potential in combination therapies.

- Histotripsy induces typical ICD by promoting the release of CRT, HMGB1 and HSP70/90, forming an in situ vaccine effect.
- It remodels tumor immune cell infiltration by increasing CD8 + T cells, NK cells, and DCs while reducing Tregs.
- It reshapes the cytokine microenvironment via upregulating pro-inflammatory factors and downregulating inhibitory cytokines.
- It triggers CD8 + T cell-mediated abscopal effect through type I interferon signaling and ferroptosis pathways.

Clinically, the #HOPE4LIVER trial confirmed a 95% technical success rate of histotripsy for liver tumors with good safety. In combination therapies, histotripsy synergizes with immune checkpoint inhibitors, targeted drugs, and chemotherapy to enhance antitumor effects by improving immune activation and tumor microenvironment.

### 3.1. Characteristics of included clinical studies

The key characteristics of the included clinical studies on histotripsy are summarized in Table 1.

**Table 1:** Characteristics of included clinical studies.

Study	Year	Study design	Tumor type	Key outcomes
#HOPE4LIVER trial	2025	Single-arm prospective	Primary/metastatic liver tumors	95% technical success rate; 7% major complication rate
Uysal et al	2025	Case report	HCC (bridge to transplantation)	Complete necrosis in treatment zone
Ziemlewicz et al	2025	Single-arm pivotal trial	Liver tumors	1-yr favorable survival and safety

HCC = hepatocellular carcinoma.

### 3.2. Immunomodulatory effects of histotripsy

The core immunomodulatory effects of histotripsy on the tumor microenvironment are listed in Table 2.

**Table 2:** Immunomodulatory effects of histotripsy.

Immune index	Change after histotripsy	Biological significance
CRT, HMGB1, HSP70/90	Upregulated	Induce ICD and in situ vaccine
CD8 + T cells, NK cells, DCs	Increased infiltration	Enhance antitumor immune response
Tregs	Decreased	Relieve immunosuppression
IFN-γ, TNF-α, CXCL10	Upregulated	Promote immune cell activation and recruitment
TGF-β	Downregulated	Reduce immunosuppressive microenvironment

CRT = calreticulin, CXCL10 = C-X-C motif chemokine ligand 10, DCs = dendritic cells, HMGB1 = high mobility group box 1, IFN-γ = interferon-γ, NK Cells = natural killer cells, TGF-β = transforming growth factor-β, TNF-α = tumor necrosis factor-α.

### 3.3. Combined therapeutic strategies of histotripsy

The synergistic mechanisms and evidence of histotripsy combined with other therapies are summarized in Table 3.

**Table 3:** Combined therapeutic strategies of histotripsy.

Combined therapy	Synergistic mechanism	Clinical/preclinical evidence
Immune checkpoint inhibitors	ICD-induced antigen release + upregulated PD-L1/CTLA-4	Enhanced abscopal effect; overcome immunotherapy resistance
Antiangiogenic drugs (lenvatinib)	Vascular normalization + improved immune infiltration	Potential conversion therapy for unresectable HCC
Chemotherapy (cisplatin, cyclophosphamide)	Immunogenic cell death + Treg depletion	Enhanced antitumor efficacy and reduced toxicity

CTLA-4 = cytotoxic T-lymphocyte-associated protein 4, HCC = hepatocellular carcinoma, ICD = immunogenic cell death, PD-L1 = programmed death-ligand 1.

## 4. Antitumor immune regulation mechanisms of histotripsy

### 4.1. “In situ vaccine” effect

Histotripsy forms an “in situ vaccine” effect by inducing ICD, which is its core immune regulation mechanism involving the release of key molecules and activation of signaling pathways.^[9,10]^

At the molecular level, Histotripsy can induce programmed necrosis (necroptosis) of tumor cells. Early after treatment, HMGB1 release and necroptosis appear in the ablation area, accompanied by ICD transcriptional responses and innate immune activation of myeloid cells and NK cells.^[11]^ qRT-PCR analysis shows that TNF-α transcription in tumors is significantly upregulated 3 days after treatment, and the colocalization of pRIPK3 and pMLKL is increased in cells at the junction of the ablation zone and the non-ablation zone, indicating the activation of the necroptosis pathway.^[11]^

CRT exposure is an early marker of ICD. One day after Histotripsy treatment, CRT translocates from the endoplasmic reticulum to the cell membrane, promoting antigen presentation.^[1]^ Nuclear translocation of HMGB1 is also a key feature of ICD,^[12]^ and the level of circulating HMGB1 in serum is significantly increased 7 days after treatment.^[1]^ In addition, the supernatant of cells treated with Histotripsy contains heat shock proteins such as HSP70 and HSP90, which form complexes with antigenic peptides and activate CD8 + T cells through the cross-presentation pathway.

### 4.2. Changes in immune cell infiltration patterns

Histotripsy can significantly reshape the immune cell infiltration pattern of the tumor microenvironment, involving coordinated changes in multiple immune cell subsets.^[13]^

Innate immune responses are activated early after treatment, with rapid infiltration of CD11b + myeloid cells, Ly6G + neutrophils, and NK cells into tumors, which is closely related to the release of DAMPs.^[11]^ Adaptive immune responses are initiated in the middle stage: the number of CD8 + T cells, CD4 + T cells, and B cells increases, regulatory T cells (Tregs) decrease, the ratio of CD8 + T cells to Tregs increases, and T cells exhibit a CD44hiCD62Llo activated phenotype with enhanced migration and killing capabilities.^[1]^ In the late stage, continuous immune cell infiltration and functional maturation are observed: the infiltration of CD8 + T cells is spatiotemporally consistent with the ferroptosis characteristics of cancer cells, and this effect can be enhanced by CTLA-4 blockade.^[11]^

The activation and maturation of DCs are key links. After treatment, the number of cDC1 and cDC2 in tumor-draining lymph nodes increases, and the activated phenotype of CD8 + T cells is enhanced. Moreover, the supernatant of cells treated with Histotripsy can stimulate immature DCs to highly express CD80 and CD86 and enhance IL-12 secretion.^[1,11]^ Meanwhile, Histotripsy can reprogram tumor-associated macrophages (M2 type) into pro-inflammatory M1 type, and this polarization switch is related to the increase of pro-inflammatory signaling molecules such as TNF. The number of NK cells increases significantly with enhanced cytotoxic activity, playing an important role in immune responses.^[11]^

### 4.3. Remodeling of the cytokine microenvironment

Histotripsy can profoundly reshape the cytokine network of the tumor microenvironment, forming a microenvironment conducive to antitumor immunity.^[14]^

Among pro-inflammatory cytokines, interferon-γ (IFN-γ) is the most significantly upregulated, with its level increasing by approximately 2-fold.^[15]^ It not only has direct antitumor activity but also activates antigen-presenting cells, enhances MHC molecule expression, and induces PD-L1 expression on tumor cells, providing targets for immune checkpoint inhibitors. The transcriptional level of tumor necrosis factor-α (TNF-α) is significantly increased 3 days after treatment, which is related to the initiation of necroptosis. It can directly induce tumor cell death, activate the NF-κB signaling pathway, and enhance vascular permeability to promote immune cell infiltration.^[11]^

Among chemokines, CXCL10 expression is significantly upregulated, which promotes the recruitment of CXCR3+/CD8 + T cells through the CXCL10-CXCR3 axis, enhancing T cell activation and effector functions. In terms of immune regulatory factors, the expression of transforming growth factor-β (TGF-β) is reduced, which relieves its inhibition on the activation of T cells and NK cells, reduces Treg differentiation, and creates conditions for antitumor immune responses.^[14]^

This cytokine remodeling has spatiotemporal specificity: the “inflammatory response” pathway is significantly upregulated in treated tumors, while the “cellular response to type I interferon” pathway is more prominently activated in tumors with abscopal effect,^[11]^ suggesting differences in immune response mechanisms between local and distant sites.

### 4.4. Mechanism of the “abscopal effect”

The “abscopal effect” induced by Histotripsy is a core proof of its immune activation ability, reflecting the systemic antitumor immune response triggered by local therapy.^[15]^

In terms of cell death mechanism, the abscopal effect is closely related to ferroptosis.^[16]^ In the non-ablation zone and distant tumors treated with Histotripsy, the accumulation of 4-HNE, a characteristic metabolite of ferroptosis, is observed in the area infiltrated by CD8 + T cells,^[17]^ and in vitro experiments have confirmed that CD8 + T cells treated with Histotripsy can mediate the accumulation of 4-HNE in tumor cells.^[11]^ At the level of immune cell trafficking, delayed HMGB1 release exists in both treated and distant tumors, which is spatially related to CD8 + T cell infiltration, and the early mobilization of NK cells may be the reason for the rapid growth inhibition of distant tumors.^[11]^

At the molecular level, the type I interferon signaling pathway plays a core role. The Z-score of the “cellular response to type I interferon” pathway in distant tumors with abscopal effect is as high as 15.5 (FDR = 2.3 × 10^^-11^, *P* = 1.8 × 10^^-14^).^[11]^ Type I interferon can activate the innate and adaptive immune systems, promote antigen presentation, and T cell activation. Adaptive immune activation is a key link: Histotripsy can release immunogenic tumor antigens, inducing the production and expansion of tumor-specific T cells.^[1]^ In addition, Histotripsy eliminates the hypoxic state in tumors, inhibits HIF1α expression, improves the functional environment of immune cells, and also supports the abscopal effect.

## 5. Research progress on combined treatment strategies of histotripsy

### 5.1. Combined application with immune checkpoint inhibitors

The combination of Histotripsy and immune checkpoint inhibitors is a highly promising therapeutic strategy, producing synergistic antitumor effects by integrating local ablation and immune activation.^[18,19]^

In terms of combination with CTLA-4 inhibitors, multiple studies have confirmed that Histotripsy can enhance their efficacy in mouse models of melanoma and hepatocellular carcinoma, especially overcoming treatment resistance in tumors with low immunogenicity such as hepatocellular carcinoma,^[1]^, and significantly improving the growth inhibition effect on contralateral tumors. Research on the combination with PD-1/PD-L1 inhibitors has shown that single use is ineffective for large-volume tumors, but after pretreatment with Histotripsy, immune checkpoint inhibitors can significantly delay tumor growth and improve the survival rate.

At the molecular level, the synergistic effect originates from 3 aspects: Histotripsy induces ICD to release a large number of tumor-associated antigens and DAMPs, providing abundant antigen sources for T cells^[1]^; increases the expression of MHC molecules on the surface of tumor cells, improving antigen presentation efficiency; and the induced inflammatory response upregulates the expression of immune checkpoint molecules such as PD-L1, providing targets for inhibitors. In terms of clinical transformation, some patients in the #HOPE4LIVER trial who received combined immune checkpoint inhibitor therapy showed a 95% technical success rate and good safety. Clinical trials specifically targeting this combined strategy are being designed, and results are expected to be announced within 2 to 3 years.

### 5.2. Combined application with targeted therapy

The combination of Histotripsy and targeted therapy mainly focuses on the field of antiangiogenesis, integrating local ablation, immune activation, and vascular normalization effects to provide new ideas for liver cancer treatment.

Among antiangiogenic drugs, lenvatinib is a representative combined drug. As a multi-target tyrosine kinase inhibitor, it can inhibit targets such as VEGFR and FGFR, and has definite efficacy in liver cancer treatment. Although there is no direct clinical data, based on the immune activation characteristics of Histotripsy and the vascular normalization effect of lenvatinib, their combination has synergistic potential.

At the mechanism level, antiangiogenic drugs can improve the abnormal structure of tumor blood vessels, increase oxygen supply and drug delivery efficiency, and enhance the therapeutic effect of Histotripsy; vascular normalization can reduce hypoxia-induced immunosuppression, creating conditions for immune activation; at the same time, it inhibits tumor cell proliferation and migration, forming a complement to the direct killing effect of Histotripsy. Individualized treatment is the core of this combined regimen. For potentially resectable patients, the conversion resection rate is significantly improved after receiving lenvatinib combined therapy, suggesting that the regimen should be formulated based on the patient’s molecular characteristics and tumor biological behavior. This strategy is suitable for patients who cannot tolerate surgery, and related preclinical studies and clinical trials are underway, with preliminary results expected within 3 to 5 years.

### 5.3. Combined application with chemotherapy

Although there are few relevant reports, based on the immunomodulatory properties of chemotherapeutic drugs and the immune activation ability of Histotripsy, their combination has unique advantages and clinical value.

In terms of drug selection, cisplatin is a key chemotherapeutic drug of interest. It inhibits replication and induces apoptosis by forming cross-links with purine bases in DNA strands and has good efficacy when combined with doxorubicin and other drugs in transcatheter arterial chemoembolization.^[20]^ In the design of combined regimens, the pretreatment with low-dose cyclophosphamide is worthy of attention. It can selectively deplete regulatory T cells, creating conditions for subsequent immune activation. This strategy has been confirmed effective in relevant intratumoral immunotherapy studies and is speculated to be applicable to Histotripsy combined therapy.

The synergistic mechanisms include the immunomodulatory effect of some chemotherapeutic drugs (such as low-dose cyclophosphamide) to enhance antitumor immune responses; induction of ICD, which overlaps with the effect of Histotripsy; and increased expression of MHC molecules and co-stimulatory molecules on the surface of tumor cells, improving antigen presentation efficiency. In clinical application, the noninvasive nature of Histotripsy allows patients to recover quickly and receive subsequent chemotherapy, and it can improve tumor drug delivery, reduce chemotherapy dosage, and adverse reactions. Attention should be paid to the differences in the immune effects of chemotherapeutic drugs, and priority should be given to drugs with immune activation or regulatory effects, and the timing and dosage of use should be optimized.

### 5.4. Specific application research for liver cancer

As the third leading cause of cancer-related deaths worldwide, liver cancer has witnessed significant progress in the treatment with Histotripsy, especially showing unique advantages in hepatocellular carcinoma (HCC).^[7]^

In terms of hepatocellular carcinoma treatment, Histotripsy has been approved by the FDA for all liver tumors.^[21]^ The #HOPE4LIVER clinical trial included 49 liver tumors from 44 patients, with a technical success rate of 95%, significantly exceeding the preset target, and the incidence of major surgery-related complications was about 7%, which is within the safe range.^[20]^ Mechanism studies have shown that in orthotopic liver cancer models of immunocompetent rats, the immune infiltration of CD11b+, CD8 + T cells, and NK cells is significantly increased after Histotripsy treatment, and the abscopal effect is more obvious in the Hepa1-6 hepatocellular carcinoma model.^[22,23]^ In terms of clinical transformation, UNOS has listed Histotripsy as a treatment option for exception candidates for liver transplantation in hepatocellular carcinoma in 2024. Bridge therapy cases have shown complete necrosis in the treatment area with no residual tumor tissue.^[24]^

In terms of combined treatment strategies, based on the successful experience of the “T + A” regimen combined with interventional therapy, researchers are exploring the combined mode of Histotripsy with atezolizumab + bevacizumab, expecting to produce a stronger synergistic effect. Individualized treatment is particularly important, and the regimen should be formulated according to the patient’s Child-Pugh classification, tumor stage, expression of molecular markers, etc, to improve efficacy.^[25,26]^

## 6. Challenges and optimization directions of combined treatment

### 6.1. Management of immune-related adverse events

The core of safety management in the combination of Histotripsy and immunotherapy is the prevention and treatment of immune-related adverse events (irAEs). irAEs result from excessive activation of the immune system. 32.8% of patients will experience various irAEs, with a median onset time of 24 weeks, mainly grade 1 to 2 reactions, and the incidence of grade 3 to 4 adverse reactions is 45%.^[27]^ Their essence is organ dysfunction caused by the immune system mistakenly attacking normal tissues.

In terms of clinical manifestations, irAEs are diverse and systemic. The most common is hypothyroidism, followed by hepatitis and colitis, and it also includes rash, pruritus, diarrhea, and adrenal insufficiency, etc.^[27]^ Hierarchical management adopts a standardized system: grade 2 reactions require oral corticosteroid treatment until remission or improvement to grade 1; grade 3 reactions may require hospitalization and intravenous high-dose corticosteroids, and retreatment may be considered in rare cases.^[28]^

In terms of prevention, although there are no specific measures for Histotripsy combined therapy, based on the experience of immune checkpoint inhibitor therapy, a comprehensive baseline assessment should be performed before treatment, and close monitoring of symptoms and laboratory indicators should be conducted during treatment, especially in the first 12 weeks; patients with a history of autoimmune diseases should be handled with caution. The treatment follows the principles of “early identification, rapid intervention, and individualized treatment.” Mild to moderate adverse reactions are treated with supportive care, while severe reactions require immediate discontinuation of immunotherapeutic drugs and the use of corticosteroids or biological agents (such as infliximab) to control inflammation. Attention should be paid to the fact that the strong inflammatory response induced by Histotripsy may overlap with immunotherapy, increasing the risk of adverse reactions, so a complete monitoring system needs to be established.

### 6.2. Development of efficacy-predictive biomarkers

In the context of precision medicine, the development of biomarkers is of great significance for the individualized implementation of Histotripsy combined therapy. As a minimally invasive biomarker, circulating tumor DNA (ctDNA) can detect genetic changes and tumor burden, providing early indications for treatment response and recurrence. The #HOPE4LIVER trial has collected patients’ blood samples for ctDNA analysis, and its dynamic changes may be more sensitive than traditional imaging in evaluating efficacy.

In terms of imaging prediction, acoustic cavitation emission signals can effectively predict treatment effects. By analyzing parameters such as final bubble lifetime, the positive and negative predictive values for predicting a necrosis rate > 95% are 0.91 and 0.89, respectively,^[29]^ which can real-time monitor the treatment process and optimize parameters. Among immune-related biomarkers, traditional indicators such as tumor mutational burden, microsatellite instability (MSI), and PD-L1 expression may be applicable to predict the effect of combined therapy. Tumors with high tumor mutational burden and MSI-H have higher immunogenicity, and the expression level of PD-L1 is related to the sensitivity to T cell attack, which helps to screen suitable patient groups.

Future biomarker development should move towards multi-omics integration, integrating information from genomics, transcriptomics, and other levels, and combining ctDNA, imaging features, and immune biomarkers to construct a comprehensive prediction model, improving prediction accuracy.

### 6.3. Formulation of individualized treatment strategies

Individualized treatment is the core of Histotripsy combined therapy, which needs to integrate factors such as the patient’s clinical characteristics, tumor biological behavior, and treatment response. Patient selection adopts a multidisciplinary tumor board (MDT) evaluation model, covering experts from multiple fields such as liver transplantation surgery, oncology, and interventional radiology. Suitable patients include those who are not suitable for surgery due to severe comorbidities or limited liver function, unresectable liver tumor patients who are unresponsive to existing treatments, and hepatocellular carcinoma patients who need tumor downstaging or bridge therapy.^[30]^

The evaluation of tumor characteristics should focus on size, location, number, and biological characteristics: tumors smaller than 3 cm can usually be treated in a single session; lesions in the dome of the diaphragm or deep in the caudate lobe may be difficult to target due to ultrasound beam limitations; multiple tumors require consideration of staged treatment or combination with other methods. The design of treatment regimens should be individualized: potentially resectable patients adopt Histotripsy combined with targeted therapy to achieve downstaging and conversion resection; advanced unresectable patients use Histotripsy combined with immunotherapy to control progression and improve quality of life.

The optimization of treatment timing and dosage is also crucial. Sequential therapy (e.g., Histotripsy first to induce immune activation, followed by immune checkpoint inhibitors) may be superior to concurrent therapy. It is necessary to balance Histotripsy treatment parameters and drug dosage to avoid toxicity accumulation. Efficacy monitoring requires the establishment of a complete follow-up system and timely adjustment of the regimen through regular imaging examinations and laboratory tests.

### 6.4. Challenges and opportunities in clinical transformation

The clinical transformation of Histotripsy faces multiple challenges: technically, anatomical location limitations (impact of bone tissue, intestinal gas, and depth) and long treatment time (the entire surgical process takes 2–4 hours) restrict the application in some patients; in terms of clinical evidence, the #HOPE4LIVER trial is a single-arm prospective study without a control group, with a small sample size and limited follow-up, requiring larger-scale randomized controlled trials to verify efficacy^[31]^; in terms of cost-effectiveness, the high cost of equipment and treatment and the scarcity of global equipment limit its promotion in developing countries; in terms of regulatory policies, differences in approval requirements in different regions and the lack of clinical application guidelines affect the global promotion of the technology.

At the same time, there are significant transformation opportunities: the technical advantages bring clear market demand, and its noninvasive and immune activation characteristics are suitable for patients who cannot tolerate traditional treatments; the prospect of combined therapy is broad, and the synergistic application with new immunotherapies and targeted therapies is expected to break through the bottlenecks of existing treatments; international cooperation is advancing rapidly. The Chinese University of Hong Kong has introduced Asia’s first Histotripsy 2.0 system and expanded application research,^[32]^ helping to promote technology and accumulate evidence; the integration of artificial intelligence, robotics, and new imaging technologies will further improve the precision, reproducibility, and safety of treatment.

### 6.5. Limitations of this review

This narrative review has several limitations. First, we performed only qualitative narrative synthesis and did not conduct a quantitative meta-analysis, owing to the substantial heterogeneity across the included studies. Second, standardized tools were not applied to assess the risk of bias in the included literature. Third, the majority of the clinical evidence included was obtained from small-scale single-arm studies, and the overall level of evidence remains to be strengthened.

## 7. Summary and outlook

### 7.1. Summary of major research findings

This review systematically analyzes the latest progress of Histotripsy in the field of antitumor immune regulation and combined therapy. In terms of technical principles, as a noninvasive, nonionizing, and nonthermal ablation technology, Histotripsy achieves mechanical tissue fragmentation through acoustic cavitation, with the core advantage of preserving antigen immunogenicity while destroying tumor tissues, inducing a strong ICD to form an “in situ vaccine” effect.^[1]^

In terms of immune regulation mechanisms, Histotripsy exhibits multi-level immune activation capabilities: at the cellular level, it induces the infiltration and activation of immune cells such as CD8 + T cells and NK cells; at the molecular level, it reshapes the cytokine network, upregulates pro-inflammatory factors such as IFN-γ and TNF-α, and downregulates immune suppressive factors such as TGF-β; and it can produce a CD8 + T cell-mediated ferroptosis-related “abscopal effect.”^[11]^

In combined treatment strategies, the combination with immune checkpoint inhibitors shows the most significant synergistic effect, which can convert “cold tumors” into “hot tumors”^[1]^; the combination with antiangiogenic targeted therapy is based on the synergistic logic of vascular normalization and immune activation; the combination with chemotherapy can enhance efficacy by virtue of the immunomodulatory effect of chemotherapeutic drugs. In terms of clinical transformation, the #HOPE4LIVER trial confirms its good safety and effectiveness, the FDA has approved it for liver tumor treatment, and it has been put into clinical application in many places around the world.^[20,33]^

### 7.2. Analysis of clinical transformation prospects

Histotripsy has broad clinical transformation prospects: in terms of indication expansion, although it is currently only approved for liver tumors, preclinical studies have shown that it is effective against a variety of solid tumors. Data from the #HOPE4LIVER trial suggest that it is effective for colorectal liver metastases, pancreatic cancer, etc,^[34,35]^ and the #HOPE4KIDNEY trial is evaluating the therapeutic effect on kidney tumors, which is expected to be expanded to more solid tumors in the future.^[36,37]^

The optimization of combined treatment regimens is the core direction. With the deepening understanding of immune activation mechanisms, the combination with new immune checkpoint inhibitors, bispecific antibodies, CAR-T cell therapy, etc, may produce revolutionary effects, and individualized combined regimens based on the concept of precision medicine will become the mainstream. The technical platform will be continuously improved: the integration of artificial intelligence and robotics will achieve more precise tumor localization and parameter optimization, and new ultrasound contrast agents and imaging technologies will further improve treatment efficacy and safety. The clinical application model will continue to innovate: its noninvasive nature makes it suitable for outpatient treatment and repeated treatment, and new models such as “fractionated treatment” and “sequential treatment” will provide more options for patients.

### 7.3. Suggestions for future

Future research should focus on the following directions: in mechanism research, further clarify the specific signaling pathways and regulatory networks of immune responses, focusing on exploring the interaction between ferroptosis and immune responses, the mechanism of the abscopal effect, and the differences in immune responses among different tumor types, while paying attention to the impact of Histotripsy on immune suppressive cells such as Tregs.^[38]^

In combined treatment research, conduct more preclinical and clinical trials to verify the efficacy of different combined regimens, clarify the optimal combined mode, timing, dosage, and course of treatment, and maximize efficacy while minimizing toxicity. In biomarker development, improve the multi-dimensional prediction and monitoring system, verify the reliability of biomarkers such as ctDNA and acoustic cavitation emission signals, and construct a comprehensive prediction model integrating multi-omics information.

In technical improvement, optimize the performance of the Histotripsy system, improve treatment efficiency, expand treatment scope, enhance image guidance and real-time monitoring capabilities, develop technical solutions to overcome acoustic barriers, and optimize immune activation effects through parameter adjustment. In clinical research, carry out multi-center, randomized controlled phase III clinical trials and real-world studies, accumulate long-term efficacy and safety data, and provide high-level evidence for clinical application. Strengthen global scientific research cooperation and technical exchanges, and improve the accessibility of the technology through technology transfer and talent training to benefit more patients.

As a revolutionary tumor treatment technology, Histotripsy has great potential in antitumor immune regulation and combined therapy. With in-depth research and technical improvement, it is expected to become an important means in the field of tumor treatment, bringing new therapeutic hope to patients.

## Acknowledgments

The authors thank the project for its support in analyzing the literature for this review. All authors declare no conflict of interest related to the research content of this paper.

## Author contributions

**Conceptualization:** Fanbo Wang.

**Data curation:** Xv Sun.

**Formal analysis:** Xv Sun.

**Funding acquisition:** Xv Sun.

**Investigation:** Xv Sun.

**Methodology:** Xv Sun.

**Project administration:** Xv Sun.

**Resources:** Xv Sun.

**Software:** Xv Sun.

**Supervision:** Xv Sun.

**Validation:** Xv Sun.

**Visualization:** Xv Sun.

**Writing – original draft:** Xv Sun.

**Writing – review & editing:** Xv Sun.
